# Concerns voiced by patients and GPs’ responses during psychosocial visits in primary care: a historical cross-sectional study

**DOI:** 10.1186/s12875-014-0188-3

**Published:** 2014-11-25

**Authors:** Ligaya Butalid, Peter FM Verhaak, Sandra van Dulmen, Jozien M Bensing

**Affiliations:** NIVEL, Netherlands Institute for Health Services Research, Utrecht, The Netherlands; Department of General Practice, Faculty of Medical Sciences, University of Groningen, Groningen, The Netherlands; Department of Primary and Community Care, Radboud University Nijmegen Medical Center, Nijmegen, The Netherlands; Department of Health Sciences, Buskerud University College, Drammen, Norway; Department of Psychology, Faculty of Social and Behavioural Sciences, Utrecht University, Utrecht, The Netherlands

**Keywords:** Doctor-patient relations, General practice, Cues, Empathy, Psychosocial factors

## Abstract

**Background:**

In a recent study comparing psychosocial consultations prior to and after the implementation of national clinical guidelines in the Netherlands, we found that general practitioners (GPs) showed less empathy in the more recent consultations. As a consequence, patients possibly have less scope to express their worries. The objective is to investigate whether patients have become more reluctant to open up about their concerns during psychosocial consultations and how GPs respond.

**Methods:**

Consultations from previous study samples videotaped between 1977 and 2008 and categorized by GPs as ‘completely psychosocial’ were selected for the present study. These consultations were observed using the Verona Coding Definitions of Emotional Sequences (VR-CoDES) to capture cues and concerns expressed by patients and GPs’ immediate responses. We compared consultations prior to (N = 121) and after (N = 391) introduction of national clinical guidelines in the 1990s.

**Results:**

In 92% of the consultations, patients presented at least one worry. These were most often expressed implicitly. However, the proportion of consultations containing at least one explicit concern changed from 24% to 37% over time. The increased number of expressed cues and concerns was partly explained by a change in GP characteristics; the latter sample contained more female and more experienced GPs. Furthermore, cues and concerns were more often expressed during later phases of consultations in recent years.

**Conclusions:**

Our study shows that patients have become somewhat more explicit in expressing their worries. However, GPs need to be aware that, still, most worries are expressed implicitly and that new concerns may appear towards the end of consultations.

## Background

In a recent comparison between consultations prior to and after the implementation in the 1990s of national clinical guidelines in the Netherlands, general practitioners (GPs) showed less empathy during more recent consultations considered psychosocial by GPs as compared to similar consultations from the 1980s [1]. There has been a shift over time towards greater emphasis on structured questioning, giving information or advice, and providing less emotional support by showing empathy [1,2]. In recent years, an integrative approach of understanding patients within their personal contexts seems to have been replaced by a more evidence-based approach characterized by active symptom exploration (see Table 1). As a consequence, patients may feel less inclined to share their worries.

**Table 1:** **The changing context of discussing psychosocial problems in primary care**

**Approach**	**Main focus**	**Background**
Integrative	*What is the meaning of the illness for the patient?*	In 1959, the Dutch College of General Practitioners stated that general practice care had to be continuous, integrative and personal.^1^ Under this agreement, GP care was explicitly placed in a broader societal and emotional context and not limited to a biomedical framework. In the years that followed, and more specifically during the 1970s and 1980s, there was greater emphasis on the importance of understanding patients within their personal contexts and GPs were encouraged to let patients talk freely during consultations. Rogers’ client-centered approach^2^ - in which empathy and unconditional positive regard were keywords - was used as a framework for dealing with psychosocial problems in general practice.
Evidence-based	*How can the illness of the patient be defined?*	From the 1990s, more emphasis was placed on evidence-based medicine with the introduction of clinical guidelines in Dutch general practice. These guidelines mostly emphasized active symptom exploration by GPs. In 1994, the Dutch College of General Practitioners published the national clinical guideline for depression.^3^ Today, there are eight clinical guidelines specifically for psychological problems and the use of guidelines is widely implemented in general practice in the Netherlands.^4^

^1^Nederlands Huisartsen Genootschap, Commissie Wetenschappelijk Onderzoek (1959). *Woudschotenrapport: Rapport over de taak van de huisarts, de zogenaamde Woudschotenmaterie [Woudschoten report: Report on the role of the general practitioner].*Utrecht:NHG.

^2^Rogers CR. (1961). *On becoming a person. A therapist’s view of psychotherapy.* Boston: Houghton Mifflin.

^3^Van Marwijk, H.W.J., Grundmeijer, H.G.L.M., Bruerer, M.M., Sigling, H.O., Stolk, J., Van Gelderen, M.G., Vintges, M., Eizenga, W.H., Burgers, J.S. (1994). NHG-standaard Depressie [Clinical guideline depression of the Dutch College of General Practitioners]. *Huisarts en Wetenschap, 37,* 482–490.

^4^Nederlands Huisartsen Genootschap (2013). *NHG-standaarden.* Retrieved from: https://www.nhg.org/nhg-standaarden.

To adequately handle psychosocial problems, GPs rely on the expression of emotions by their patients. Most expressed emotions relate to psychosocial issues such as depressive feelings, stress or concerns about life changes [3]. However, patients are likely to express implicit cues to underlying emotions and worries, rather than voicing emotional problems explicitly and spontaneously [4]. These implicit cues are often vague and ambiguous and require further exploration by the GP and empathy from GPs may encourage patients to be more open and direct in further expressions of their worries [5]. Addressing and responding to patients’ emotions and psychosocial issues early on during consultations may reduce the tendency for patients to wait to express remaining problems or concerns until the closing stages of the visit [6].

Because of the previously found reduction in empathy showed by GPs, new questions arise regarding the role of patients during consultations. It can be argued that patients’ manner of communicating has changed over time like GPs’ communication styles have. Patients may have become less explicit in sharing their concerns, which could explain the decrease in empathy by GPs. Despite a broad consensus on the importance of activating patients and increasing their autonomy in health care [7], patients do not seem to be as participatory as expected during consultations [2]. Moreover, studies on the closing phase of general practice consultations show that patients often present ‘doorknob’ concerns [6]. It can be argued that if patients have indeed become more reluctant to share their worries in recent years, they may postpone expressing their concerns until later phases of the consultations. On the other hand, the emphasis put on active symptom exploration with the introduction of Dutch clinical guidelines in general practice may motivate GPs to actively invite patients to share their concerns regarding psychosocial problems during the earlier, diagnostic, phase of consultations.

### Aims of the study

We aim to study the role of patients during psychosocial consultations prior to and after the introduction of guidelines on psychological disorders in Dutch general practice in the 1990s. We decided to further investigate previously examined consultations that were considered psychosocial by GPs [1], and focus on whether patients share their worries and how GPs respond to these worries.

First, given the previously found decline in empathy by GPs over time, we expect to find a decline in expression of concerns or cues to underlying concerns by patients during recent consultations. Second, we aim to explore the timing of expressed worries of patients during consultations from the two time periods. Greater reluctance among patients to share their worries may also imply postponement of expressing concerns. On the other hand, active symptom exploration may motivate GPs to invite patients to share concerns during diagnostic phases of consultations. Last, we expect that GPs have become less responsive and inviting to patients’ worries.

## Methods

### Videotaped consultations

Dutch general practice consultations were videotaped in the period from 1977 to 2008 as part of previous studies on doctor-patient communication [8-13]. Participating GPs were followed for at least a full day (or a series of consecutive days). An unmanned camera was placed in the consultation room to record consultations. A research assistant was present in the waiting room to ask patients’ informed consent for participating in the study and answer any additional questions. All participating GPs and patients provided some general background information in a basic questionnaire (e.g. age, gender, assessment of psychosocial aspects), which enabled the selection of consultations for additional observational research in the present study. We included consultations from six previous study samples (1977–1980, 1982–1984, 1989, 1995, 2000–2001, 2007–2008) and assigned these samples to the two periods of interest (prior to clinical guidelines: 1977–1980, 1982–1984, 1989, versus after the introduction of guidelines: 1995, 2000–2001, 2007–2008). The total database of available videotaped consultations consisted of 5,184 consultations (1,895 in the first period, 3,289 in the second period). First, the GP from each videotaped consultation assessed the degree to which psychosocial aspects determined the consultation (1 = completely somatic; 2 = mainly somatic; 3 = both somatic and psychosocial; 4 = mainly psychosocial; 5 = completely psychosocial). Second, we selected consultations assessed by the GP as being ‘completely psychosocial’. GPs assessed 150 consultations in the first period and 394 consultations in the second period as psychosocial. Owing to deterioration in the technical quality of some videotaped consultations, we excluded 31 consultations (28 in the first period, 3 in the second period) and one consultation was excluded because patient characteristics (e.g. age) were not available. Our analyses were conducted on 512 consultations (121 in the first period, 391 in the second period) and we specified whether psychological (N = 185), social (N = 62) or physical (N = 265) symptoms were presented by patients during these consultations by using the International Classification for Primary Care (ICPC) codes that were available for all consultations in the database.

The studies were carried out in accordance with Dutch privacy legislation. The privacy regulations were approved by the Dutch Data Protection Authority. According to Dutch legislation, approval by a medical ethics committee was not required for these observational studies. All participating physicians and patients gave their informed consent.

### Measures of communicative behaviour

Patient cues and concerns were coded using the Verona coding definitions of emotional sequences, VR-CoDES-CC [14]. A cue is defined as ‘a verbal or non-verbal hint which suggests an underlying unpleasant emotion but lacks clarity’ (*“I cannot stand it anymore”*), while a concern is ‘a clear and unambiguous expression of an unpleasant current or recent emotion where the emotion is explicitly verbalized’ (*“I feel very anxious”*). GPs’ immediate responses (lag 1) to cues and concerns were coded using VR-CoDES-P [15]. We only coded lag 1 responses, which refers to the first utterance after a voiced cue or concern, while excluding delayed responses (e.g. lag 2 or 3). Responses were coded according to two major conceptual factors: explicitness (explicit versus non-explicit responses) and space provision for further disclosure of the cue of concern (space-providing versus space-reducing responses). See Table 2 for definitions and examples of the response categories.

**Table 2:** **Response categories by GPs according to VR-CODES-P**

**Non-explicit, reducing space**		
Ignore	No reference is made whatsoever to the concern	
Shutting down	Denying patient’s concern	“Oh don’t be silly”
Information advice	Giving information or advice in a way that does not open space for further disclosure	“Headaches are very common”
**Non-explicit, providing space**		
Silence	Silence to invite patient to talk about the concern	
Back channel	Minimal prompt to invite patient to talk about the concern	“Hmm” “Ok…”
Acknowledgement	Implicit comment beyond the minimal back channel	“I can see that”
Active invitation	Clearly inviting, but implicit in relation to the concern	“Would you like to tell me more?”
Implicit empathy	Expression of feeling or understanding, without explicit reference to the concern	“It must be hard”
**Explicit, reducing space**		
Switching	Response that changes the frame of reference of the concern	“Did you have similar symptoms in the past?”
Postponing	Reducing space for talking about the concern at this moment	“I would like to talk with you about this in a minute”
Information advice	Acknowledging concern, but giving information or advice that does not open space for further disclosure	“You do not need to worry, headaches are very common”
Active blocking	Mentioning concern and explicitly refusing to talk about it	“Worrying does not do you any good”
**Explicit, providing space**		
Content acknowledgement	Echoing, reflecting back, giving paraphrases or summarizing content of concern	“You’ve been experiencing headaches for a week now”
Content exploration	Asking about content	“How long have you’ve been experiencing headaches?”
Affect acknowledgement	Echoing, reflecting back, giving paraphrases or summarizing emotional aspects of concern	“You’re worried”
Affect exploration	Asking about emotional aspects	“Why are you worried?”
Empathy	Expression of feeling or understanding, with explicit reference to the concern	“I understand that the pain is worrying you”

Approximately 10% of the observed consultations were coded by the two coders involved in this study. The interrater reliability of the VR-CoDES-CC and VR-CoDES-P was found to be satisfactory to good. We calculated an intraclass correlation (ICC) of 0.56 for concerns and 0.89 for cues. The mean intraclass correlation of the GPs’ response categories was 0.71 (range 0.43 - 0.86).

Total visit duration was timed in seconds for all videotaped consultations in the database. We timed the initial opening statements by patients, which starts after GPs solicitations (*“What can I do for you today?”)* and ends when GPs initiate the next phase of exploration by asking either open-ended (*“Tell me more about the head aches”*) or closed-ended questions (*“Is it worse in the morning or in the evening?”)*. The last phase of the consultation can be considered the therapeutic phase of the consultation and is characterized by GPs giving information and advice. The interrater reliability of the durations of the different phases during consultations (initial opening statements, exploration including physical examination, and giving information and advice) was found to be good. The mean intraclass correlation of the duration of the phases was 0.93 (range 0.90 – 0.99).

### Statistical analyses

To account for the variation in communication skills between GPs, we used multilevel models with random intercepts (multilevel Poisson regression analysis for count variables). The multilevel models consisted of consultations (level 1) nested within GPs (level 2). The number of consultations per GP in the sample varied between 1 and 15. However, since 80% of the GPs had five or less consultations included in the present study, we could not calculate or report on intraclass correlations. We included dummy variables for both periods (1977–1989 versus 1995–2008) and examined estimated frequencies for the three types of symptoms (psychological, social and physical symptoms). First, we used multilevel Poisson regression models to estimate frequencies of communication categories by GPs per consultation. In these analyses, we included duration of consultation, patient characteristics and GP characteristics as centred covariates. Second, based on these estimates we tested whether there were differences in communication categories between the two periods.

## Results

### Consultation characteristics

Mean duration of consultations in the second period (1995–2008) was significantly longer than during than in the first period (see Table 3). When comparing the patient and GP characteristics between the two time periods, we found that patients and GPs were significantly older in 1995–2008 compared to 1977–1989. The gender ratios of patients did not differ significantly between the two periods, but the percentage of female GPs was higher in the second period (31% versus 7%).

**Table 3:** **Characteristics of the study sample (consultations considered completely psychosocial by GPs)**

**Patient and consultation characteristics**	**1977-1989 (N = 121)**	**1995-2008 (N = 391)**	Comparison*
*Consultation duration*	mean (sd)	mean (sd)	
Duration	11 min,13 sec	14 min,55 sec	***t*** (510) = 5.03***
*Age*	mean (sd)	mean (sd)	
Years	38.2 (16.0)	44.6 (17.7)	***t*** (510) = 3.59***
*Gender*	N (%)	N (%)	
Male	45 (37%)	128 (33%)	Chi^2^ (1) = 0.82
**GP characteristics** ^**†**^	**1977-1989 (N = 42)**	**1995-2008 (N = 162)**	
*Age*	mean (sd)	mean (sd)	
Years	40.6 (7.4)	47.3 (6.5)	***t*** (200) = 5.76***
*Gender*	N (%)	N (%)	
Male	39 (93%)	111 (69%)	**Chi** ^**2**^ (1) = 10.15**
*Professional experience*	mean (sd)	mean (sd)	
Years working as a GP	12.3 (7.0)	17.7 (8.4)	***t*** (174) = 3.69***

*Analyzed with T-tests for continuous variables (consultation duration, age, professional experience) and Pearson’s Chi^2^ for categorical variables (gender). *p <0.05, **p <0.01, ***p <0.001.

^†^Age and working experience was missing for 2 GPs in the period 1977–1989. Working experience was missing for 26 GPs in the period 1995–2008. These data could not be recovered.

### Expression of cues and concerns by patients in both periods

In 92% of the consultations in this study, patients presented at least one cue or concern. The proportion of consultations containing at least one implicit cue did not differ between the two periods. However, the proportion of consultations containing at least one explicit concern was larger in the second period (see Table 4).

**Table 4:** **Voiced cues and concerns by patients in both periods**

		**1977-1989 (N = 121)**	**1995-2008 (N = 391)**	**Chi** ^**2**^ *
		**N (%)**	**N (%)**	
Consultations with at least one cue		106 (88%)	363 (93%)	3.29
Consultations with at least one concern		29 (24%)	144 (37%)	6.8**
Timing of cues and concerns:				
Cues and concerns during initial statements		138 (15%)	442 (11.5%)	8.64**
Cues and concerns during exploration phase		337 (36.5%)	1,168 (30%)	13.67***
Cues and concerns during therapeutic phase		449 (48.5%)	2,259 (58.5%)	29.11***
Estimated frequencies of cues and concerns per consultation^†^		Estimate (95% CI)	Estimate (95% CI)	Chi^2^
Model 1: complete model^‡^	Psychological	7.7 (6.1 - 9.6)	9.9 (8.8 - 11.1)	3.57
	Social	9.5 (7.5 - 12.1)	10.2 (8.7 - 11.9)	0.22
	Physical	6.0 (4.7 - 7.6)	5.7 (5.1 - 6.4)	0.10
Model 2a: without patient characteristics^§^	Psychological	7.8 (6.2 - 9.8)	9.6 (8.6 - 10.8)	2.48
	Social	9.7 (7.6 - 12.3)	10.3 (8.8 - 12.0)	0.16
	Physical	5.7 (4.5 - 7.3)	5.9 (5.2 - 6.6)	0.05
Model 2b: without GP characteristics^||^	Psychological	7.7 (6.2 - 9.5)	10.0 (8.9 - 11.1)	4.69*
	Social	8.9 (7.1 - 11.1)	10.2 (8.8 - 11.9)	1.01
	Physical	5.9 (4.7 - (7.4)	5.8 (5.2 - 6.4)	0.03

*Significant Chi^2^-tests indicate significant differences between the two periods, *p <0.05, **p <0.01, ***p <0.001.

^†^Estimated with multilevel Poisson regression models.

^‡^Included covariates: consultation duration, age of patient, gender of patient, age of GP, gender of GP.

^§^Included covariates: consultation duration, age of GP, gender of GP.

^||^Included covariates: consultation duration, age of patient, gender of patient.

When looking at the timing of the cues and concerns expressed by patients, we see that the percentage of cues and concerns voiced during initial statements and exploration was higher in the first period compared to the second period. Patients more often expressed cues and concerns during the last phase of information and advice in more recent consultations (see Table 2).

Patients expressed on average 8.64 cues and 0.80 concerns per consultation. Because of the low frequencies of explicit concerns, we calculated estimated frequencies of voiced cues and concerns combined. When comparing these frequencies between the two periods, while controlling for patient characteristics and GP characteristics, we did not find significant changes. Since patient characteristics and GP characteristics differed between the two periods, we checked whether frequencies of expressed cues and concerns changed when running our models without the patient characteristics or the GP characteristics as covariates. Running the model without patient characteristics did not change our findings. However, we found that in the model without GP characteristics the number of cues and concerns in consultations involving psychological symptoms differed significantly between the two periods (Chi-square = 4.69, *p* < .05). This indicates that differences in GP characteristics between the two study samples account for differences in the number of cues and concerns expressed by patients.

### GPs’ responses to cues and concerns in both periods

In both periods, GPs’ responses to cues and concerns were mostly characterized by giving space for patients to talk about their concerns in a non-explicit way (see Table 5): being silent or giving minimal responses (back channels) were the most frequent ways for GPs to respond to cues and concerns. We did not find changes in response categories by GPs when comparing the two periods.

**Table 5:** **Estimated frequencies of responses to cues and concerns by GPs**

		1977-1989* ^**†**^	**1995-2008**	**Chi** ^**2**^^**‡**^
		**(N = 121)**	**(N = 391)**	
		**Estimate (95% CI)**	**Eestimate (95% CI)**	
Non-explicit, reducing space	Psychological	0.3 (0.2 - 0.6)	0.6 (0.4 - 0.8)	3.16
	Social	0.5 (0.2 - 0.8)	0.5 (0.3 - 0.8)	0.01
	Physical	0.6 (0.4 - 1.1)	0.4 (0.3 - 0.5)	3.04
Non-explicit, providing space	Psychological	4.4 (3.4 - 5.8)	5.7 (5.0 - 6.6)	2.80
	Social	6.1 (4.6 - 8.1)	6.7 (5.5 - 8.1)	0.24
	Physical	3.4 (2.5 - 4.5)	3.3 (2.9 - 3.8)	0.00
Explicit, reducing space	Psychological	1.0 (0.7 - 1.4)	1.2 (0.9 - 1.4)	0.47
	Social	0.8 (0.5 - 1.2)	0.8 (0.6 - 1.2)	0.03
	Physical	0.8 (0.5 - 1.1)	0.7 (0.6 - 0.8)	0.20
Explicit, providing space	Psychological	1.4 (1.0 - 2.1)	1.7 (1.4 - 2.1)	0.69
	Social	1.5 (1.0 - 2.2)	1.8 (1.3 - 2.5)	0.58
	Physical	0.7 (0.5 - 1.2)	1.0 (0.8 - 1.2)	1.55

*Estimated with multilevel Poisson regression models.

^†^Included covariates: consultation duration, age of patient, gender of patient, age of GP, gender of GP.

^‡^Significant Chi^2^-tests indicate significant differences between the two periods.

## Discussion

Our study shows that patients are likely to express their worries during primary care psychosocial consultations and that the proportion of consultations with at least one explicit concern was higher during more recent consultations. The number of expressed cues and concerns was partly explained by a change in GP characteristics; the latter sample contained more female GPs and more experienced GPs. Furthermore, we found that late-arising concerns were common in our study sample and were even more likely during more recent consultations. We found that GPs responded mostly by non-explicit communicative behavior, such as being silent or giving minimal responses to indicate that they were listening, but we did not find indications that GPs reduced the space for emotional disclosure.

### Patients’ expressiveness

In contrast to our expectations, we did not find a decrease in cues and concerns expressed by patients over time. Patients have become more familiar with psychosocial problems such as anxiety and depression and therefore may be more willing to open up about their concerns. Information on mental health has become readily available, for example through the Internet. Internet users seem to have high levels of mental health literacy and are well able to answer knowledge-based questions regarding psychological disorders [16]. Interestingly, although the proportion of explicitly expressed concerns increased over time, the number of expressions during consultations did not change. Patients’ expressiveness has become somewhat more explicit, but not more extensive over time.

Patients tend to share their worries most often during the therapeutic phase as opposed to the initial or exploration phase of consultations. Late arising cues and concerns were more likely in recent consultations, compared to older consultations. With the introduction of clinical guidelines in the 1990s, more emphasis is placed on giving information and advice [1,2]. It is important that GPs are aware that providing new information to patients may also elicit new questions and concerns.

### Facilitating communication by GPs

We found that the number of expressions of cues and concerns was partly dependent on GP characteristics. GPs in the second sample (1995–2008) were older, had more professional experience, were more often female, and were more likely to evoke more expressions of cues and concerns by patients. This is in line with previous research that shows that patients are more likely to share psychosocial issues and feel more empowered when talking to female GPs [17]. Accordingly, feminization of Dutch general practice care may partly account for changes in the way GPs facilitate communication about emotional issues.

Moreover, the GPs in our study were not likely to respond in an explicit way to patients’ concerns, either by showing empathy or by further exploration of the worries voiced. This indicates that GPs remain relatively passive listeners, rather than actively providing emotional support for their patients. Studies in psychiatric settings show similar findings; physicians often hesitated to respond and were likely to respond with passive listening skills as opposed to engaging in active emotion-focused skills [18]. In some situations indirect responses to patients’ emotions may be less intrusive and more adequate than explicit responses. However, active solicitation of concerns by GPs can prevent late arising, or even unvoiced, concerns [6]. Recent versions of clinical guidelines for depression and anxiety published in 2012 refer to the importance of empathy during consultations involving these psychological problems [19]. We consider this allusion to the importance of empathy in clinical guidelines as a positive development.

### Strengths and limitations of the study

One of the strengths of this study is that we used the concerns and cues of patients as the starting point for our observations and analyses and focused on the patient’s role. Furthermore, we examined consultations using videotaped real-life general practice consultations over a thirty-year period, enabling a comparison of doctor-patient communication prior to and after the introduction of national clinical guidelines in the 1990s. Video recording is a valid method of examining doctor-patient communication: the influence of the video recorder on participants is marginal [20] and the participants were unaware that the analyses would focus on psychosocial problems.

A possible weakness of the study is that we did not include consultations in which GPs were unable to recognize or identify psychosocial issues during consultations. We decided to only include consultations assessed by GPs as completely psychosocial, since we believe that cues and concerns by patients are most likely to be clearly expressed in these types of consultations. However, to be able to generalize the results to other health problems there is a need for replication studies within other consultation types, which may include more hidden psychosocial problems. Another limitation of our study is that we did not specify the nature and content of expressed cues and concerns by patients. Therefore, we do not know for each specific expressed cue or concern whether the patient was referring to psychosocial issues. However, due to the low frequency of the cues and concerns voiced, we decided to not further categorize these as this would complicate the interpretation of quantitative analyses and we could not guarantee interrater reliability. Furthermore, we only looked at immediate responses (lag 1) of GPs after patients’ expressed cues and concerns; we did not include GPs’ delayed or random empathic responses. This may explain why the previously found decline in empathy - assessed over the entire consultation - was not found in the present study. We decided to look at lag 1 responses following expressed worries because we were interested in GPs immediate responsiveness; it can be argued that empathic responses are most appropriate and supportive when following directly after patients’ expressed worries.

## Conclusions

The aim of our study was to look at patients’ expressed worries and GPs’ immediate responses during psychosocial consultations in Dutch general practice. In earlier analyses, we found an increase in the proportion of consultations assessed by GPs as being mainly or completely psychosocial, while they gave less room for disclosing emotion-related issues.^1^ In the present study, we see that patients have become somewhat more open in expressing their concerns in recent years. We found that expression of worries is partly dependent on GP characteristics; experienced and female GPs were more likely to evoke more expressions of worries compared to less experienced and male GPs. With the changing GP population, the likelihood of expressing worries by patients seems to have changed.

While patients seem to have become somewhat more explicit in sharing their worries, GPs tend to respond mostly implicitly to these concerns. We argue that GPs should be encouraged to also respond explicitly to patients concerns, in order to ensure that patients feel heard and understood. Moreover, GPs need to be aware that concerns can appear throughout the consultation; towards the therapeutic phase of the consultation, new questions and concerns may arise, which also deserve full attention and appropriate responses from GPs.

## Abbreviations

GP: General practitioner
GPs: General practitioners
VR-CoDES-CC: Verona coding definitions of emotional sequences - cues and concerns
VR-CoDES-P: Verona coding definitions of emotional sequences - provider’s responses
ICPC: International Classification for Primary Care
ICC: Intraclass correlation
